# Organoids Modeling Stroke in a Petri Dish

**DOI:** 10.3390/biomedicines12040877

**Published:** 2024-04-16

**Authors:** Chiara Giorgi, Vanessa Castelli, Michele d’Angelo, Annamaria Cimini

**Affiliations:** Department of Life, Health and Environmental Sciences, University of L’Aquila, 67100 L’Aquila, Italy; chiara.giorgi2@graduate.univaq.it (C.G.); vanessa.castelli@univaq.it (V.C.)

**Keywords:** brain organoids, ischemic stroke, drug screening, reperfusion, central nervous system

## Abstract

Stroke is a common neurological disorder, the second leading cause of death, and the third leading cause of disability. Unfortunately, the only approved drug for it is tissue plasminogen, but the therapeutic window is limited. In this context, preclinical studies are relevant to better dissect the underlying mechanisms of stroke and for the drug screening of potential therapies. Brain organoids could be relevant in this setting. They are derived from pluripotent stem cells or isolated organ progenitors that differentiate to form an organ-like tissue, exhibiting multiple cell types that self-organize to form a structure not unlike the organ in vivo. Brain organoids mimic many key features of early human brain development at molecular, cellular, structural, and functional levels and have emerged as novel model systems that can be used to investigate human brain diseases including stroke. Brain organoids are a promising and powerful tool for ischemic stroke studies; however, there are a few concerns that need to be addressed, including the lack of vascularization and the many cell types that are typically present in the human brain. The aim of this review is to discuss the potential of brain organoids as a novel model system for studying ischemic stroke, highlighting both the advantages and disadvantages in the use of this technology.

## 1. Introduction

Stroke is the second leading cause of death and the third leading cause of disability; it was the cause of 11.6% of deaths in 2019, and from 1990 to 2019, the absolute number of cases increased with a 70.0% rise in incident strokes and a 43.0% increase in deaths from stroke [1,2]. Stroke is a neurological deficit caused by an acute focal injury of the central nervous system (CNS) by a vascular cause; among strokes, ischemic stroke is the most frequent, representing 62.4% of strokes worldwide. Ischemic strokes are caused by a reduction of blood flow, generally caused by arterial occlusion [2,3]. Ischemic stroke is characterized by neurological death leading to disability in adult life [2]. There are several lifestyles risks, such as smoking, unhealthy diets, and high blood pressure, and these are increasing due to the aging of population [2,4]. The symptoms of ischemic stroke are the following: facial palsy, arm and leg motor impairment, head deviation, aphasia; and agnosia. These can be absent, mild, moderate, or severe [5]. Women usually have a higher risk of stroke over their lifespan than men, but, since they have more atypical risk factors, symptoms, and less awareness, they have a lower chance of being diagnosed and of being treated for strokes than men. Some of women’s risk factors are oral contraceptives, hormone use, pregnancy, and menopause [2]. Once the patient has been suspected of having an ischemic stroke, some parameters must be evaluated; among them there are the heart rate and rhythm, fever, blood pressure, and endocarditis. Later, to identify the stroke area, the patient should undergo brain imaging via computed tomography (CT) or Magnetic resonance imaging (MRI). The former is usually the first choice; the latter is more sensitive but is less used because it takes longer than CT and it is less available [4].

Ischemic stroke damage is caused by a lack of oxygen and nutrient supply to the affected brain region. This causes a cascade of events leading to cell damage and the loss of neurological function; among these events, there are inflammation, apoptosis, and oxidative stress [6]. Moreover, additional damage could be caused after oxygen reperfusion. The increasing level of oxygen can cause the formation of reactive oxygen species (ROS), the matrix metalloprotease degradation of the blood–brain barrier (BBB), and glutamate excitotoxicity [3].

To date, there are few FDA-approved drugs or non-drug treatments. The most used ones are the tissue plasminogen activator (t-PA) and endovascular thrombectomy (EVT), and these have therapeutic time windows of 3/4.5 h and 6 h, respectively [7,8]. Moreover, 3% to 5% of patients are eligible for tPA treatment because of its narrow therapeutic window and certain contraindications [7]. So, it is important to find new therapies and drugs, even if in recent years the new therapies that have been studied have failed. One of the reasons why they are failing is the differences between the animals used in research and human patients. Rodents are the most used animals in research, but differences have been found between rodents and humans in the response of neurons, astrocytes, and microglia after oxygen–glucose deprivation, meaning this could impact stroke research [9]. It is also important to note that rodents also have a different brain anatomy and organization, meaning that they cannot perfectly mimic the human brain. One of the differences is represented by the percentage of white matter, which is lower in rodents, which is important for the prognosis of stroke outcome [7].

Traditional bidimensional (2D) cell cultures have been used to model hypoxic-ischemic injuries, but they have several limitations. Even if they have been used to study different cell types in brain diseases, have a lower cost, present fewer ethical issues than animal models, and can be used in high-throughput studies, they cannot be used to study the more complicated features of disease development. For example, in the study of ischemic stroke, perfusion is crucial, but vascularization cannot be mimicked in 2D cell cultures [10].

For these reasons, it is necessary to find new models to study ischemic strokes, and a possible tool could be represented by brain organoids. Organoids are three-dimensional (3D) cell-culture models that can be generated from human stem cells. Organoids contain cell types able to self-organize so that they can mimic a specific organ [11]. For example, via human-induced pluripotent stem cells (hiPSCs), it is possible to create brain organoids via patient-derived neurons to study disease mechanisms [12]. Because the 3D environment of organoids resembles the tissue and organ of interest, they are a valuable tool for disease modelling, overcoming both interspecies differences and 2D-model limitations [13].

Based on the reported evidence, this review aims to discuss the potential of brain organoids as a novel system for studying ischemic stroke, highlighting both the advantages and disadvantages in the use of this 3D technology.

## 2. Ischemic Stroke

**Figure 1:**
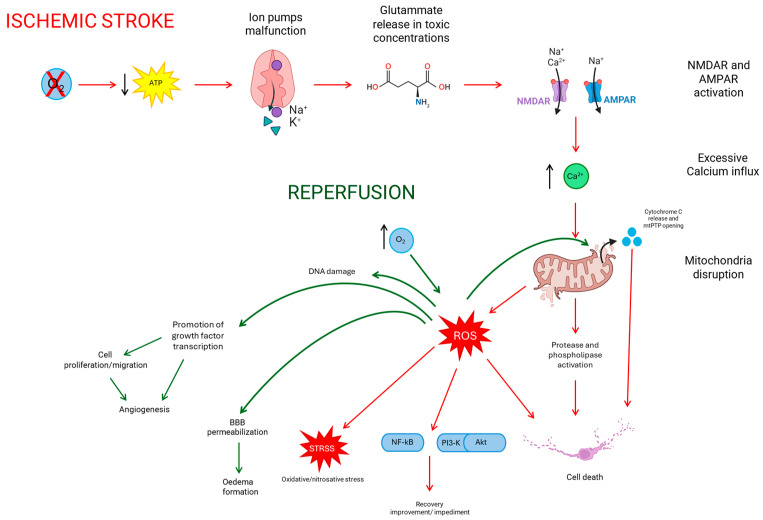
Schematic representation of the pathophysiology of ischemic stroke/reperfusion.

During an ischemic stroke, a lack of oxygen and nutrients causes an impairment in cell metabolism, leading to a dysfunction in energy-dependent processes [14]. The resulting loss of energy causes an ionic imbalance and neuronal depolarization with a consequent inhibition of neurotransmitter reuptake and neurotransmitter release [15,16]. Furthermore, the reduction of ATP production and consequent ion pumps’ malfunction result in cellular acidification, promoted by a shift to anaerobic glycolysis [17]. Sodium–potassium ATPase is one of the affected ionic pumps [15]. One of the excitatory neurotransmitters that is released in toxic concentrations is glutamate. Its excessive release activates the ionotropic N-methyl-D-aspartic acid (NMDA) and 1-amino-3-hydroxy-5-methyl-4-isoxazole propionic acid (AMPA) receptors, causing an excessive calcium influx [15,18]. This intracellular calcium influx triggers signaling cascades that result in mitochondrial disruption, caused by mitochondrial permeability transition pore (mtPTP) opening and cytochrome c release, and the activation of free radicals, phospholipases, and proteases that degrade structural proteins, cell membranes and nucleic acids, ending with cellular death [14,15,18]. Free radicals are responsible for the Phosphatidylinositol 3-kinase (PI3-kinase)/serine/threonine kinase(Akt) pathway activation and nuclear factor kappa B(NF-kB) activation, which can both cause recovery improvement or impediment [16]. Moreover, free radicals cause oxidative and nitrosative stress. These processes are accelerated during reperfusion with an increase in oxygen leading to lipid peroxidation and DNA damage [19]. Another kind of damage caused by oxidative stress is due to the release of molecules that trigger an inflammatory response, which, in turn, activates brain-intrinsic microglia and an increased blood–brain barrier permeability with consequent immune-cell infiltration [20]. See Figure 1 for a schematic representation of the pathophysiology of ischemic stroke and reperfusion.

### 2.1. Treatments

Stroke treatment consists of the reperfusion of the affected brain area, which can be performed via intravenous thrombolysis or endovascular thrombectomy (EVT). Intravenous thrombolysis is performed using an intravenous recombinant human tissue plasminogen activator (IV tPA): alteplase. It is the only Food and drug administration (FDA)-approved treatment [4,21], and it converts plasminogen into plasmin, which cleaves the cross-link connections between fibrin molecules, dissolving so the thrombus is responsible for ischemic stroke [3,22]. Fibrin molecules are blood clots’ scaffolds, and once they are dissolved they produce fibrin-degradation products; these are later degraded by other enzymes [22]. IV tPA is most effective if it administered within 90 min from the symptoms’ onset, but it can still be beneficial within 3/4.5 h [23]. Because of the numerous contraindications, possible complications, and the small therapeutic window, tPA is administered only to 3–5% of the patients [24]. Among the tPA complications there are hemorrhages, which can intensify stroke injuries, and, more rarely, orolingual angioedema, which in the worst scenario can affect airway patency [23]. To date, tPA remains the only FDA-approved thrombolytic agent, even if more studies have been conducted on the use of Tenecteplase, a bioengineered variant of tPA which has shown a lower risk of hemorrhage, a longer half-life, and more specificity with fibrin [23,25].

EVT is the only FDA-non-drug-approved method for ischemic stroke treatment, and its therapeutic window is about 6 h [8]. It has been demonstrated that EVT has a better functional outcome than tPA, and it is associated with a better outcome in the case of acute large vessel occlusion and large ischemic core than standard medical therapy; moreover, the administration of tPA before EVT can significantly improve the outcome of patients compared to the ones who received only a single treatment [5,26,27].

### 2.2. Reperfusion

As mentioned before, reperfusion can result in some adverse effects [17,18]. After the oxygen influx is restored, there is the formation of ROS inside the cells. Their presence causes DNA, protein, and lipid damage, mtPTP opening with consequent intracellular calcium influx, and the activation of inflammatory cascades [28]. It is known that due to the apoptosis and necrosis of brain cells after ischemia, the inflammatory system is stimulated, leading to more reperfusion injuries [29]. More damage is caused by ROS [30], and one of their effects is to increase the permeability of the vessels in the BBB that enhance the probability of the formation of an oedema [31]. BBB integrity is correlated with the severity of the patient’s outcome; the BBB permeabilization with the oedema and ROS damages can lead to the hemorrhagic transformation of ischemic infractions, which is one of the most severe consequences of ischemic stroke, influencing what type of treatment a patient can receive [29].

Despite all these complications due to reperfusion, it remains beneficial. Indeed, ROS can also promote the transcription of growth factors and, consequently, cell proliferation and migration. All of this process causes vascular remodeling/angiogenesis, helping to reduce the damage [17].

Due to its possible adverse effects, is it necessary to find neuroprotective strategies for ischemia and reperfusion as well as to extend the time window of treatments [18]. Brain organoids could represent a good model for ischemic stroke studies. See Figure 1 for a schematic representation of the pathophysiology of ischemic stroke and reperfusion.

## 3. Brain Organoids

The term “organoid” refers to cells that proliferate in a 3D manner, forming clusters; these cells can self-organize and differentiate into functional cell types with the ability to recapitulate the structure and function of an organ in vivo. For these reasons, organoids must have some specific characteristics; like the 3D structures with all the cell type present in the organ to be modelled, they must also mimic the organ functions and they should contain all the cell kinds found in the organ [32,33]. The self-organization ability of the cells composing the organoid is also due to the controlled environment in which they grow. These cells are able to self-organize via cell-sorting processes that are possible thanks to the activation of various signaling pathways mediated by intrinsic cellular components, the extrinsic environments, and the media provided [34].

### 3.1. Organoid Culture and Modeling

**Figure 2:**
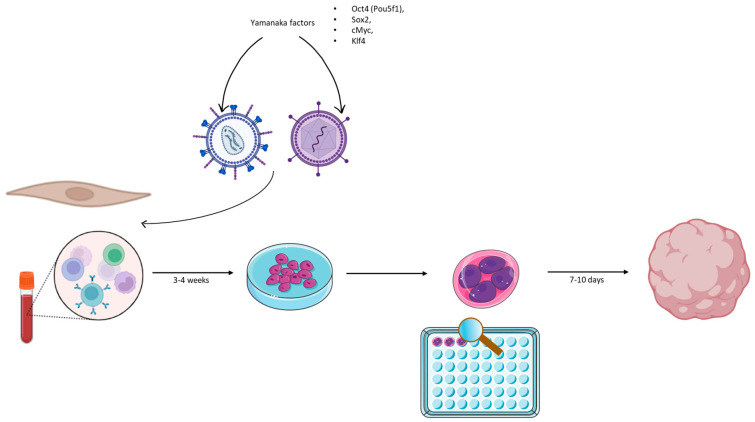
This figure summarizes the process that leads to the formation of brain organoids, starting with the collection of fibroblasts or peripheral blood mononuclear cells (PBMCs) from patients and their reprogramming into hiPSCs via Yamanaka’s factors, introduced via retrovirus or lentivirus-mediated gene transduction. After 3–4 weeks, cells have been reprogrammed to hiPSCs and their culture can start; after few days, there is the formation of the embryoid bodies, and in 10 days there is the formation of brain organoids that require maturation and that can be used for experiments [38,39].

Organoids usually derive from the differentiation of embryonic stem cells (ESC) or hiPSCs. These cells have pluripotent properties that allow for the generation of the three germ layers, self-renewal processes, and differentiation that enables them to organize into an organ-specific pattern. To allow these cells to differentiate, they are exposed to various growth/inhibitor factors that usually have a role in gastrulation and organogenesis [34,35]. Moreover, both ESC and hiPSCs can derive from patients that express a disease or from heathy donor. For these reasons, organoids are a valuable tool for studying disease, and it has been demonstrated that, thanks to cell manipulation, fibroblasts from microcephalic patients could be reprogrammed into hiPSCs to study the mechanism of this disease. It is possible to genetically manipulate hiPSCs to express autistic features to study non-idiopathic autism spectrum disorder [36,37]. See Figure 2 for a brief description of the organoid culture process starting from fibroblast/PBMCs reprogramming.

hiPSCs can be induced to mimic the steps and differentiation processes that lead to the formation of embryonic germ layers; the 3D structures formed by these steps are called embryoid bodies (EBs). These are aggregates that spontaneously form the three germ layers [40] and can be created by both avoiding contact with cells with a plastic support or using a scaffold that represents an extra cellular matrix (ECM). Scaffolds can be biological, such as Matrigel, which derives from mouse sarcoma cells, or synthetic. If scaffold techniques are used, the EB and organoids are cultured with the help of spinning bioreactors [34].

### 3.2. Brain Organoids

Human brain organoids, thanks to the presence of the same cell types typical of the human brain, have the ability to reproduce different brain regions that can interact with each other, or they can be programmed to resemble just one specific region of the brain [41,42,43]. The human brain starts its development as a neuronal tube, and then shows, via cellular and molecular process, a mature organization structure that has three main regions: the forebrain, the midbrain, and the hindbrain [44,45]. This particular organization is not present in brain organoids, but the different cell classes that compose organoids are organized in a multilaminar way, making them similar to the human brain [36,46]. Brain organoids represent the brain at up to 24 weeks of its development; but, beyond this period of time, organoids start to develop a necrotic core. This necrotic-core formation is more due to a lack of vascularization [47,48]. Over the years, new ways to overcome the lack of vascularization and oxygenation have been developed. One example is the use of spinning bioreactors as well as the use of BDNF (brain-derived neurotrophic factor) to promote neuronal survival; other methods that have been used include the transplant of brain organoids into mice brains or the slicing method, in which a sliced organoid is left floating in orbital shakers [41,46,49,50,51]. Brain organoids display inhibitory/excitatory synapses, and they also show the presynaptic vesicles, meaning that the neurons that compose them are mature; furthermore, they show a spontaneous neuronal activity [13,41,49,51]. To date, brain technology has been developed to the point where it is possible to create region-specific organoids, such as the midbrain, the hypothalamus, the blood–brain barrier, the cerebellum, and the spinal cord; these kinds of organoids are useful in the study of region-specific deficits [12,52,53,54,55]. Some research groups also developed brain-region-specific organoids that were later fused together. For example, ventral and dorsal brain organoids were fused together, showing interneuron migration from one region to another; moreover, these regions were connected synaptically and showed connection between excitatory neurons [56].

Brain organoids can be used also as a tool for the disease modelling and target identification of neurological disorders [57]. To date, different diseases have been studied with organoids, such as neurodegenerative disorders, neurodevelopmental diseases, Down’s syndrome, autism spectrum disorder, and brain tumors [37,49,58,59,60,61,62]. Furthermore, brain organoids have been used to study the differences in the cortical development of different animals (e.g., macaques and chimpanzees) and humans [63]. The presence of active neurons has been demonstrated via calcium imaging, which shows the presence of action-potential active neurons [64]. Recently, some research groups have worked to use brain organoids as new models to study ischemic stroke [7].

### 3.3. Advantages and Limitation in the Use of Organoids

For many reasons, organoids are more useful than the classical 2D cell culture and animal models. Indeed, the 2D cell culture presents some limitations, such as poor cell differentiation into specific cell types, which instead is one of the main characteristic of organoids, in which there are all the different cell types present in the reference organ [65]. One of the limitations of animal models is the impossibility of reproduction of all the human disorders; this is caused by the difference at neurological levels between the animal model brain and the human one. Unfortunately, not only does the brain of the animal model fail to represent all the complex aspects of the human brain, but due to the animal’s life span, it is also difficult to model some diseases related to aging (Parkinson and Alzheimer’s) [66]. Another advantage of organoids is that they can derive from patients’ cells, meaning it is possible to study the disease starting with cells carrying the specific disease mutation, resulting in a more representative model, and thanks to their genetic stability they are useful for carrying high-throughput screening studies [67,68,69]. Also, because organoids are representative of the brain during its development, they are useful to study the neural network of the developing human brain, human-brain development, disease etiology, and molecules to discover new therapies [70,71,72]. Moreover, working with organoids is easier than working with animals, reducing the complexity of experiments and the ethical problems; furthermore, they have less ethical, bureaucratic, and tissue-conservation-related problems than working with samples deriving from patients, which are difficult to use to obtain and undergo postmortem modifications [35,69,73].

Even if organoids are better at representing human brains than animal models and 2D cultures, they have some limitations. For example, they have typical fetal neocortex properties associated with a reduction in aging markers that can represent a problem in the study of age-related diseases. However, to overcome this problem, new protocols that involve the use of pretreatments with molecules that accelerate maturation have been developed [74,75].

The lack of oxygenation inside the organoids and the formation of a necrotic core represents additional challenges in organoid cultures. Some research groups have developed new methods to overcome these organoids’ limitations; for example, co-culture with epithelial cells, ETS Variant Transcription Factor 2(ETV2) induction via genetic engineering, the creation of 3D neuronal constructs with microglia and vasculature, and organoid slicing [76,77,78]. The variation organoids have in differentiation, morphology, and cell composition among samples of the same batch and different batches is a huge problem, but there are some ways to increase homogeneity such as in micro-scaffolds, mini spinning bioreactors, and the use of control groups for each batch to reduce the interference from the different genetic backgrounds [11,13,46,79]

## 4. Brain Organoids as a Tool for Ischemic Stroke Studies

Ischemic stroke has been modeled through the years both with animal models and with 2D cell cultures. While animal models have several limitations due to the interspecies differences, 2D cell cultures are not representative of the pathophysiological processes of the disease [9,80]. Moreover, mice models need some techniques such as slicing that may affect the result of the experiments due to the additional damage inflicted during the cutting procedures [7]. Indeed, 3D cultures, and in particular organoids, better represent the organ of reference and can demonstrate to researchers the interactions among different cell types that compose the organ. For example, because of the reduced oxygenation of the cells in brain organoids, they mimic better the in vivo microenvironment than the 2D cultures, which have a more oxygenated environment [81].

For these reasons, some research groups have studied stroke on brain organoids.

Wang and collaborators selected brain organoids at 85 days of culture to test some pro/anti-apoptotic compounds and some neuroprotective molecules against ischemic stroke to see if organoids could be used for ischemic stroke studies. They selected organoids at 85 days because they had a good Tuj1+/SOX+ ratio, making this time-point organoid similar to in vivo brains. They tried different oxygen–glucose deprivation (OGD) times and selected 8 h after checking the cell cytotoxicity of LDH release and the cell apoptosis of Caspase 3 activity. For this study, they provided organoids during OGD experiments with anti-apoptotic and pro-apoptotic compounds pan-Caspase inhibitor Z-VAD-FMK and Bcl-2 inhibitor navitoclax. The TUNEL assay (apoptotic cell detection) and the Nissl’s staining (survival neuron detection) confirmed the anti-apoptotic effect of Z-VAD-FMK and the pro-apoptotic effect of navitoclax in the brain organoids. The results showed that the Z-VAD-FMK-treated organoids had more Nissl’s positive cells in respect to vehicles, and they showed no differences to the control group. On the other hand, the group treated with navitoclax had a decreased amount of Nissl’s positive cells compared to vehicle organoids. TUNEL assay analysis showed a decrease in TUNEL-positive cells than the vehicle group and no differences to the control group in organoids treated with Z-VAD-FMK and an increase in this kind of cells in navitoclax-treated organoids with respect to the vehicle. These results showed that not only are brain organoids sensitive to ischemic stroke injuries and can be used as a reliable model, but also that they are an efficient tool for drug-screening tests due to the changes shown with the administration of pro/anti-apoptotic compounds. This research group also studied the cell-death pathways involved in ischemic stroke, looking for markers of apoptosis, necroptosis, autophagy, and ferroptosis, and, notably, OGD organoids were positive for all of them. So, they evaluated four neuroprotective compounds (edaravone, butylphthalide, P7C3-A20, and ZL006) in OGD brain organoids. The cell cytotoxicity of LDH release and the cell apoptosis of Caspase 3 activity showed that these compounds protected OGD-organoids from stroke injuries, meaning they could also be used for tests aimed to check the neuroprotective anti-stroke drug efficacy [7].

Another research group tested if extracellular vesicles (EVs) could have neuroprotective/neuroregenerative properties. EVs are a group of vesicles that contain different molecules (non-coding RNAs, DNA, and proteins) and can be divided into exosomes, macrovesicles, and apoptotic bodies. It is known that EVs from mesenchymal stem cells (MSCs) have a role in neuroprotective/neuroregenerative processes. In their study, Zheng and colleagues performed OGD for 8 h, followed by 24 h of reoxygenation and treated organoids with different concentrations of neural progenitor cell (NPC)-derived EVs. As a result, NPC-EVs reduced the cell-death rate of OGD organoids. They performed the same test on mice affected by cerebral ischemia, comparing the NPC-EVs group to mice treated with MSC-EVs. These experiments showed a reduction in post-stroke brain injury in mice treated with NPC-EVs comparable to the results obtained in MCS-EV-treated mice. More tests also showed an increase in post-stroke neuroregeneration and neurological recovery in NPC-EVs mice. This study demonstrated that NPC-EVs are effective both in vitro and in vivo experimental conditions, suggesting that EVs could be used as a therapeutic tool against ischemic stroke [82]. These results showed that brain organoids, with some improvements, are a valuable and effective tool to study new therapies for ischemic stroke and also for a better understanding of molecular mechanisms of stroke.

Iwasa and collaborators, in another study, demonstrated that vitamin digestion and absorption, fat digestion and absorption, the peroxisome proliferator-activated receptor (PPAR)-signaling pathway, and pyruvate kinase isoform M2 (PKM2) are possible key markers of neuronal cells in response to oxygen–glucose deprivation/reperfusion) OGD/R. For this purpose, they performed a transcriptome analysis on brain organoids from hiPSCs and different cell types (human hepatocellular carcinoma cell line (HepG2), human telomerase reverse transcriptase immortalized fibroblasts (hTERT-BJ), human glioblastoma cell line (U251MG), and murine microglia (BV2)). They revealed that the expression level of PKM2 of brain organoids after OGD/R was predominantly elevated while it was not changed in other cell types, suggesting a possible role of PKM2 as a hypoxic marker in brain organoids. Because gene networking showed PPAR involvement in OGD/R, and considering also the results obtained in organoids with PKM2, and that is it known that PPARγ inhibits PKM2 in breast cancer and that the PPARα/γ agonist improves stroke outcome in mice, they hypothesized that therapeutic agents related to lipid metabolism, such as the PPAR agonist, may be effective against ischemia [83].

Another research group subjected brain organoids to hypoxic conditions, OGD, and glucose deprivation. They showed that organoids that received oxygen deprivation were subjected to the disruption of neuronal cells and to size reduction, but that reoxygenation increased neuronal-cell proliferation without restoring maturation; OGD organoids were more damaged, showing a greater reduction in size than the hypoxic organoids, with a loss of layer structures. They also did not benefit from the reoxygenation phase; in contrast, glucose deprivation alone did not affect the size of the brain organoids [84]. See Table 1 for a summary of these studies.

## 5. The Use of Brain Organoids as Therapeutics against Ischemic Stroke

Brain organoids have also been used as a possible therapy against ischemic stroke. Wang and colleagues transplanted brain organoids into ischemic stroke mice. This study demonstrated that the transplant of cerebral organoids after middle cerebral artery occlusion (MCAO) reduced brain damage, improving neurological motor function, and that the cells from cerebral organoids are able to migrate towards different and distant brain regions. Moreover, they demonstrated that this transplantation enhanced neurogenesis, inhibited neuronal apoptosis, and promoted axonal regeneration [8]. Another research group transplanted brain organoids entirely or dissociated them in single cells in stroke mice. The authors reported that single cells from organoids failed to repair the infarcted area, while whole organoids survived well in the infarcted tissue, differentiated into target neurons, repaired infarcted tissue, and ameliorated animals’ motor performance [85].

## 6. Conclusions

The 3D brain organoids are able to mimic the development and main function of the human brain, giving them strong potential for drug screening and disease modeling. Their 3D structure allows researchers to overcome different problems that derive from the study of diseases with 2D structures and, being developed from hiPSCs, allow researchers to overcome the interspecies differences they found while studying diseases with animal models [86].

Nevertheless, there are still several limitations, such as the necrotic core, the lack of vascularization, and the high variability. Another important aspect that must be taken into consideration is the absence of BBB and non-neuronal cell types in organoid cultures. These are aspects that should be incorporated in organoids culture to obtain a better representation of the human brain, meaning that despite the researchers’ efforts to improve this method, brain organoids need more work [7]. Researchers are actively working to overcome these limitations and enhance the fidelity of brain organoids.

However, brain organoids, if well developed, could be a substitute for the animal models, allowing researchers to shorten the drug-screening process and to model human neurological diseases more accurately, also reducing the ethical concerns that come when animal models are used. Indeed, brain organoids represent a novel technology, not only to study the underlying mechanisms of ischemic stroke but also to test potential therapies, and, in addition, they can be used as regenerative therapeutics in stroke. Despite these advantages, it is important to note that the use of brain organoids in stroke research is still in its early stages, and there are several challenges that need to be addressed. Apart from the limitations of brain organoids, it is important to underline the lack of vascularization, and the absence of glial components, which play a key role in cerebral ischemia.

Despite these challenges, recent studies on ischemic stroke using brain organoids suggest a promising future in this research field. They offer the possibility to study new molecules for neuroprotection, neurodevelopment, and even new antioxidants that could help with reducing the negative effect of reperfusion processes. Overall, brain-organoid technology holds immense promise for advancing stroke research and improving treatment options.

## Figures and Tables

**Table 1:** This table briefly summarizes the studies that involve the use of brain organoids for studying stroke that have been discussed in this review.

Study	OGD Type	Techniques	Main Results
Testing pro/anti-apoptotic compounds (Z-VAD-FMK and navitoclax) and neuroprotective compounds (edaravone, butylphthalide, P7C3-A20, and ZL006) [7]	Oxygen/glucose deprivation for 8 h	Cytotoxicity LDH release, TUNEL assay Nissl’s staining, and Caspase 3 activity	All the compounds had the expected effect on organoids, suggesting brain organoids can be used for drug-screening experiments.
Testing possible neuroprotective/neurodegenerative properties of EV on organoids after OGD [82]	Oxygen/glucose deprivation for 8 h and 24 h of reoxygenation	Treatment with different concentrations of neural progenitor cells (NPC)-derived EVs	NPC-EVs reduced the cell death rate of OGD organoids suggesting a possible use of organoids to discover new therapy against stroke and to better understand the molecular mechanisms of stroke.
Looking for OGD/R markers on organoids [83]	Oxygen/glucose deprivation and reperfusion	Transcriptome analysis and gene networking	The expression level of PKM2 of brain organoids after OGD/R was elevated, suggesting PKM2 could be a possible hypoxic marker.
Modelling hypoxic brain injuries [84]	Oxygen/glucose deprivation, hypoxia, glucose deprivation	Analyzing organoids’ size, cell proliferation, and disruption.	OGD organoids were the most damaged and they did not benefit from reoxygenation phase.
